# Metagenomic analysis reveals the virome profiles of *Aedes albopictus* in Guangzhou, China

**DOI:** 10.3389/fcimb.2023.1133120

**Published:** 2023-06-02

**Authors:** Ye Xu, Jiabao Xu, Tong Liu, Peiwen Liu, Xiao-Guang Chen

**Affiliations:** ^1^ Department of Pathogen Biology, Institute of Tropical Medicine, School of Public Health, Southern Medical University, Guangzhou, China; ^2^ School of Basic Medical Sciences, Zhejiang Chinese Medical University, Hangzhou, Zhejiang, China

**Keywords:** *Aedes albopictus*, virome, small RNA sequencing, mosquito, virus diversity

## Abstract

**Introduction:**

*Aedes albopictus* is an aggressive invasive mosquito species widely distributed around the world, and it is also a known vector of arboviruses. Virus metagenomics and RNA interference (RNAi) are important in studying the biology and antiviral defense of *Ae. albopictus*. However, the virome and potential transmission of plant viruses by *Ae. albopictus* remain understudied.

**Methods:**

Mosquito samples of *Ae. albopictus* were collected from Guangzhou, China, and small RNA sequencing was performed. Raw data were filtered, and virus-associated contigs were generated using VirusDetect. The small RNA profiles were analyzed, and maximum-likelihood phylogenetic trees were constructed.

**Results:**

The small RNA sequencing of pooled *Ae. albopictus* revealed the presence of five known viruses, including Wenzhou sobemo-like virus 4, mosquito nodavirus, Aedes flavivirus, Hubei chryso-like virus 1, and Tobacco rattle virus RNA1. Additionally, 21 new viruses that had not been previously reported were identified. The mapping of reads and contig assembly provided insights into the viral diversity and genomic characteristics of these viruses. Field survey confirmed the detection of the identified viruses in *Ae. albopictus* collected from Guangzhou.

**Discussion:**

The comprehensive analysis of the virus metagenomics of *Ae. albopictus* in this study sheds light on the diversity and prevalence of viruses in mosquito populations. The presence of known and novel viruses highlights the need for continued surveillance and investigation into their potential impact on public health. The findings also emphasize the importance of understanding the virome and potential transmission of plant viruses by *Ae. albopictus*.

**Conclusion:**

This study provides valuable insights into the virome of *Ae. albopictus* and its potential role as a vector for both known and novel viruses. Further research is needed to expand the sample size, explore additional viruses, and investigate the implications for public health.

## Introduction

*Aedes albopictus*, originating from the forests of Southeast Asia, was first recorded on La Réunion Island in 1913 (Edwards, 1920). It has since become an aggressive invasive species, widely distributed around the world (Bonizzoni et al., 2013). *Ae. albopictus* is also a known vector of arbovirus (Delatte et al., 2008), including dengue virus (Lambrechts et al., 2010; Ferreira-De-Lima and Lima-Camara, 2018), Zika virus (Liu et al., 2017), and Chikungunya virus (Delatte et al., 2008). The Zika outbreak in 2015 emphasized the necessity to extensively study the biology of this mosquito species (De Oliveira et al., 2017).

Virus metagenomics, or virome analysis, is the study of viromes in biological samples. This suggests that the virome plays an important role in *Aedes* vector competence. RNA interference (RNAi) are conserved, sequence-specific gene regulation mechanism in eukaryotes, including insects (Blair and Olson, 2015), *Caenorhabditis elegans* (Conte et al., 2015), mammals (Cullen, 2017; Ding et al., 2018), and plants (Borges and Martienssen, 2015). In mosquitoes, RNAi regulates development and physiology, and plays an important role in anti-viral defenses (Gammon and Mello, 2015; Moon et al., 2015; Göertz et al., 2016; Airs and Bartholomay, 2017). It consists of various pathways that play a crucial role in the antiviral response in mosquitoes (Balakrishna et al., 2017). The siRNA pathway (Olson and Blair, 2015) is one such pathway that is triggered by virus-derived double-stranded RNA (dsRNA), which is then cleaved by the RNase III enzyme Dicer-2 (Dcr-2) to generate virus-specific small RNAs (vsiRNAs) that are 21 nt in length. These vsiRNAs guide the RNA-induced silencing complex (RISC) to locate and degrade complementary viral RNA sequences. Therefore, the production of 21-nt viRNA molecules can be considered a hallmark of antiviral RNAi. Mosquitoes produce small RNAs of interest after virus infection, allowing for the reconstruction of the genome of the infected virus. Next-generation sequencing is a robust technology for obtaining small RNA profiles. By combining bioinformatics tools and virus databases, it is possible to recover assembled contigs and homologous viroid sequences. In 2015, a study of the viral community in three samples, *Culex tritaeniorhynchus*, *Anopheles sinensis*, and a mixture pool of *Armigeres subalbatus* and *Culex fatigans* from Hubei province, demonstrated a high abundance and diversity of viruses (Shi et al., 2015). In 2016, metagenomics sequencing of adult *Anopheles* mosquitoes from Liberia, Senegal, and Burkina Faso found a number of virus and virus-like sequences from mosquito midgut contents (Fauver et al., 2016). In the same year, distinct virome profiles were found in mosquitoes from the Caribbean and locations on the US East Coast (Frey et al., 2016). The viral community of *Ae. albopictus* also been studied (Shi et al., 2015; He et al., 2021; Calle-Tobón et al., 2022). These studies have increased our understanding of virus diversity in mosquito vectors. Plant viruses possess the ability to engage in diverse interactions with vectors, including both non-persistent and circulative modes of transmission. Notably, there exists a paucity of evidence in prior studies indicating the capacity of *Aedes albopictus* to serve as vectors for the transmission of plant viruses.

In this study, we aimed to obtain metagenomic data by sequencing the whole small RNA from pooled *Ae. albopictus* mosquitoes from the field. We collected female adults, male adults, and larval mosquitoes from Guangzhou, and analyzed the virus-associated sequences in the small RNA profiles. Our findings revealed the presence of five viruses, including flaviviruses, that could infect *Ae. albopictus.* Furthermore, we identified 21 new viruses that had not been previously reported. This research represents the comprehensive analysis of the virus metagenomics of different sex and larval *Ae. albopictus* in a field setting.

## Materials and methods

### Mosquito collection

Mosquito samples of *Ae. albopictus* used in this study were collected between April and October 2017 from separate locations in Guangzhou (**Table 1**). The collection sites included gardens, schools, mountains, stations, and offices. Samples were collected from female adults, male adults, and larvae at each location, and were then pooled according to the develop stage. Each sample was divided into three pools, resulting in a total of three female pools (Female 1, Female 2, and Female 3), three male pools (Male 1, Male 2, and Male 3), and three larvae pools (Larvae 1, Larvae 2 and Larvae 3) for small RNA sequencing.

**Table 1:** Geographic information of samples.

Number	Name	Location	Date	Female	Male	Larvae
1	Jiahe Wanggang	N23°13′58.88″ E113°16′55.23″	2017/04/13	25	21	30
2	Baiyun Mountain	N23°10′49.43″ E113°17′30.67″	2017/04/17	50	8	30
3	Xichang	N23°08′16.50″ E113°13′51.92″	2017/05/27	40	4	0
4	Feixiang Park	N23°10′18.26″ E113°15′29.55″	2017/05/28	32	26	30
5	Shayuan	N23°05′33.69″ E113°15′13.08″	2017/06/11	27	32	30
6	Guangzhou Light Industry Technician College	N23°05′37.76″ E113°17′52.17″	2017/07/26	18	14	30
7	Yuexiu Park	N23°08′35.45″ E113°15′31.75″	2017/08/06	10	6	0
8	Yanling Park	N23°09′29.90″ E113°19′3.92″	2017/09/17	20	10	0
9	Southern Medical University	N23°11′18.44″ E113°19′42.04″	2017/10/20	30	27	30
10	Jiangxia Station	N23°12′5.78″ E113°16′29.05″	2017/09/21	14	12	0

### RNA preparation and sequencing

In order to clear the remaining residues in digestive tracts, the collected mosquitoes were fed with 10% glucose for 24 hours before processing, Prior to extraction, mosquitoes were washed three times with sterile PBS. The OSE-Y30 electric tissue grinder (Tiangen, China) was used to grind the samples with 200 μl of TRIzol reagent (Invitrogen, USA) on ice. The sample homogenate was then clarified by centrifugation at 20,000 ×g (4°C) for 30 minutes and filtered through a 0.45 μm membrane filter (Millipore, Billerica, USA). The TruSeq Small RNA Sample Prep Kit (Illumina, San Diego CA) was used to prepare the sRNA library with 1 μg of total RNA as input, following the manufacturer’s instructions. Briefly, polyacrylamide gel electrophoresis (PAGE) purification of RNA bands, enriching RNA molecules in the size range of 16–50 nt the small RNAs, were preferentially ligated with 3’ and 5’ adapters, reverse transcribed using Superscript II reverse transcriptase (Invitrogen, Carlsbad CA), and PCR amplified. During the amplification process, a unique oligonucleotide barcode sequence was incorporated into each library for multiplexing purposes. The resulting small RNA libraries were then size-selected on 2% TBE-agarose gels and purified using MinElute Gel Extraction kits (Qiagen). The purified cDNA libraries were eluted in water and quality-checked on the 2100 Bioanalyzer. The small RNA libraries were sequenced using SE50 by Illumina Nextseq 500 according to the manufacturer’s instructions. Small RNA library sequencing was completed by Genedenovo (Guangzhou, China).

### Small RNA data analysis

Raw data were filtered to remove adaptor sequences, reads shorter than 20 nt, and low-quality reads. Virus-associated contigs were generated from the clean data using VirusDetect v1.6 with the *Ae. albopictus* genome as reference sequence. Small RNA length distribution graphs and viral genomic position maps were produced using the viRome package (http://www.ark-genomics.org/bioinformatics/virome) in R. The sequences from the small RNA libraries of all nine pools were submitted to the NCBI Sequence Read Archive (BioProject - PRJNA527345). Maximum-likelihood phylogenetic trees were constructed using the Mega 7 program (Kumar et al., 2016) with 1,000 bootstrap replications.

### PCR amplification and sequencing

*Ae. albopictus* from the places listed in **Table 1** were recollected. From each place, ten female, male adults and larvae were collected. RNA from field-collected *Ae. albopictus* were extracted individually and were tested with primers (**Table 2**) designed to amplify gaps between contigs using Primer STAR GXL (Code No. R050A, TAKARA) with the following conditions: 30 cycles of 98°C for 10 seconds, 58°C for 15 seconds, and extension time at 68°C based on the length of the product, followed by a final extension at 68°C for 10 minutes.

**Table 2:** Primers used in this study.

Virus	Forward	Sequence(5’-3’)	Reverse	Sequence(5’-3’)
Wenzhou sobemo-like virus 4	WZSLV-F	TTGGCTATCACCGCGCTAAA	WZSLV-R	GGCGGCAGCACTTTGATATG
*Ae. albopictus* nodavirus	ANV-F	CCACAACCAACATTGACAGC	ANV-R	CACGGTGCTTTGATACATGG
Hubei chryso-like virus 1	HCLV-F	GTGGGAAAACGACTCTCGCC	HCLV-R	AGCACCATCGGCTATCAACC
*Aedes* flavivirus	AFV-F	GGGTACCGTTGCCTTACTGA	AFV-R	CGAGCGACAATGCTCATTTA
Tobacco rattle virus RNA1	TRV-F	AATGTGCACGCAACAGTTCT	TRV-R	TCTCTTCCTCCTACCGTCCA

## Results

### Small RNA sequencing of pooled *Ae. albopictus*

To assess the viral community associated with the pooled mosquito samples, we performed next generation sequencing of the small interfering RNAs in the *Ae. albopictus* genome. For female pools, 1,404,145 reads, 1,354,127 reads, and 1,497,473 reads were generated, respectively. For male pools, 1,992,372 reads, 1,229,666 reads, and 1,240,011 reads were generated, respectively. For larvae pools, 1,421,265 reads, 1,533,100 reads, and 1,529,231 reads were generated, respectively. Reads longer than 20 nt were counted for further analysis.

**Figure 1:**
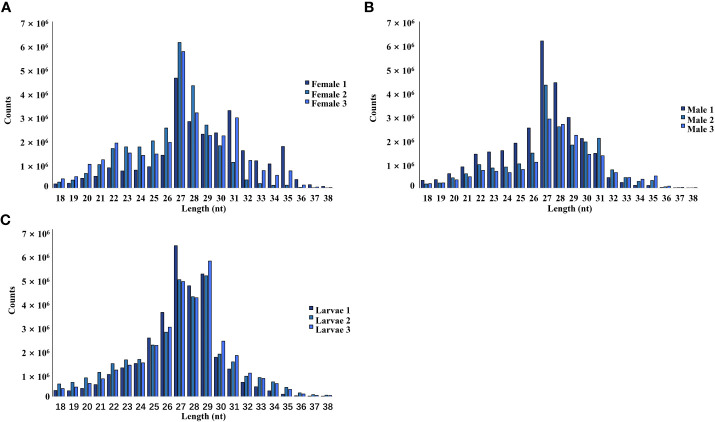
Length and size distribution of trimmed reads of the sequenced libraries from female **(A)**, male **(B)**, and larvae **(C)** samples.

The female, male, and larvae pools had a relatively high proportion of piRNA (25-30 nt), followed by siRNA (21-22 nt) and miRNA (24 nt). In this study, piRNA was the most dominant in all nine pools (**Figure 1**).

Mapping reads to the virus databases of NCBI indicated a mapping rate of 0.25-0.93% in the different pools (**Table 3**). VirusDetect (Zheng et al., 2017), a program used to efficiently identify viruses from NGS data, and viRome (Watson et al., 2013) were used to analyze the sRNA sequencing data. The information for contigs was aligned to known virus genomes of each pool and has been summarized in **Table 4**. In total, five known viruses were found and confirmed by PCR, including Wenzhou sobemo-like virus 4, mosquito nodavirus, *Aedes* flavivirus, Hubei chryso-like virus 1, and tobacco rattle virus RNA1.

**Table 3:** Summary for the reads mapping to mapping to virus genome database.

Sample		Total reads	Mapping to *Ae. albopictus* Genome		Mapping to viral genome	
	Symbol		Read counts	Mapping rate	Read counts	Mapping rate
Female_Rep1	Female 1	1,404,145	437,537	31.16%	3,580	0.25%
Female_ Rep2	Female 2	1,354,127	449,894	33.22%	4,146	0.31%
Female_ Rep3	Female 3	1,497,473	494,264	33.01%	4,897	0.33%
Male_ Rep1	Male 1	1,992,372	344,262	27.76%	6,332	0.32%
Male_ Rep2	Male 2	1,229,666	391,977	31.88%	3,945	0.32%
Male_ Rep3	Male 3	1,240,011	620,482	31.14%	4,148	0.33%
Larvae_ Rep1	Larvae 1	1,421,265	333,529	21.81%	5,904	0.42%
Larvae_ Rep2	Larvae 2	1,533,100	353,761	23.07%	8,805	0.57%
Larvae_ Rep3	Larvae 3	1,529,231	266,015	18.72%	14,169	0.93%

**Table 4:** Summary of contig hits to the virus database in nine pools.

Reference	Length	Genus	Description	Female 1	Female 2	Female 3	Male 1	Male 2	Male 3	Larvae 1	Larvae 2	Larvae 3
KX882831	2961	NA	Wenzhou sobemo-like virus 4	2941 (99.3%)	2355 (79.5%)	2861 (96.6%)	2742 (92.6%)	2729 (92.2%)	2570 (86.8%)		2181 (73.7%)	2371 (80.1%)
KJ741266	11063	*Flavivirus*	*Aedes* flavivirus		3180 (28.7%)							
GU144510	1130	*Betanodavirus*	Mosquito nodavirus						488 (43.2%)			
KX882962	2897	NA	Hubei chryso-like virus 1							2570 (88.7%)		
AF166084	6791	Tobravirus	Tobacco rattle virus RNA1									2226 (32.8%)

Contigs were assembled from the reads of nine Ae. albopictus pools from Guangzhou and used for virus identification using the VirusDetect Program. The total length of contigs and the coverage against the virus genome are shown for each sample pool.

### Wenzhou sobemo-like virus 4

**Figure 2:**
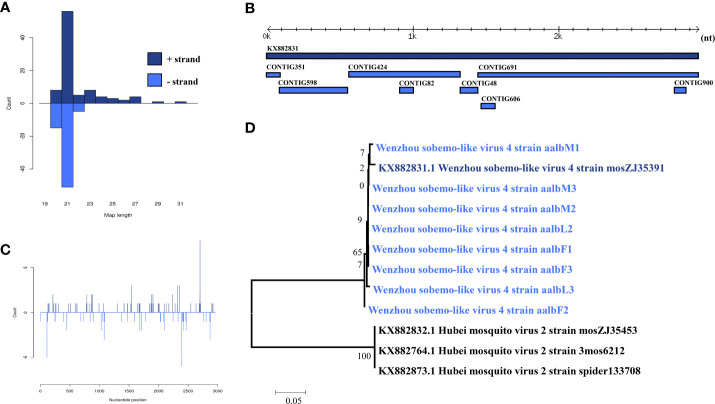
Analysis of small RNAs derived from Wenzhou sobemo-like virus 4 and the phylogenetic analysis. **(A)** alignment of assembly contigs to Wenzhou sobemo-like virus 4 (KX882831) genome. Dark blue represents Wenzhou sobemo-like virus 4 genome, and light blue represent assembled contigs. **(B)** Size distribution of sRNA derived from Wenzhou sobemo-like virus 4. **(C)** phylogenetic analysis indicates the relationship between the eight strains (light blue) and published Wenzhou sobemo-like virus 4 genome sequence (dark blue). Species in black were outgroup. **(D)** Small RNAs distribution across the genome of Wenzhou sobemo-like virus 4 in both positive and negative stains. The x-axis (1 to 3000) represents the number of reads that cover each position of the genome. The y-axis is the count.

Wenzhou Sobemo-like virus 3 was identified in eight pools. Most Wenzhou sobemo-like virus 4 mapped sRNA were 21 nt in length (**Figure 2B**), indicating that they were generated by the siRNA pathway. In the female pools, 8, 13, and 12 assembly contigs (**Figure 2A, D**) were generated, covering 79.5-99.3% of the genome with an average identity of 97%, respectively. In the male pools, 14, 14, and 15 assembly contigs were generated, covering 86.8-92.6% of the genome with an average identity of 98%, respectively. It was also found in two larvae samples with four and two assembly contigs and covering 39% and 80.1% of the viral genome with an average identity of 98.57%. The phylogenetic tree (**Figure 2C**) indicated that all Wenzhou sobemo-like virus 4 strains were closely related to the Wenzhou sobemo-like virus 4 strain mosZJ35391.

### *Ae. albopictus* nodavirus

**Figure 3:**
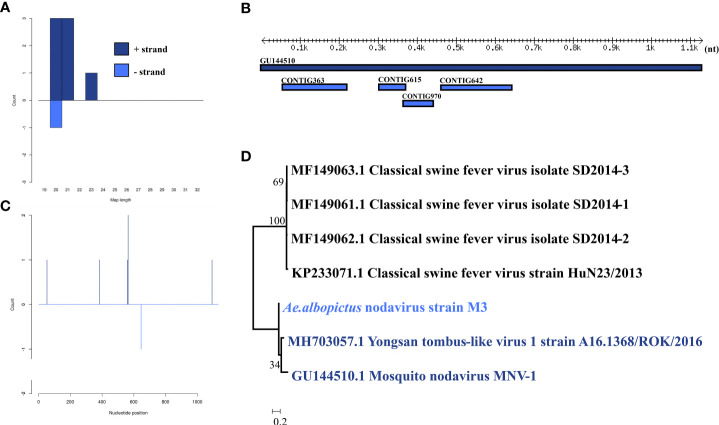
Analysis of small RNAs derived from *Ae. albopictus* nodavirus and the phylogenetic analysis. **(A)** Alignment of assembly contigs to *Ae. albopictus* nodavirus (GU144510) genome. Dark blue represents Mosquito nodavirus genome, and light blue represent assembled contigs. **(B)** Size distribution of sRNA derived from *Ae. albopictus* nodavirus. **(C)** Phylogenetic analysis indicates the relationship between the 8 strains (light blue) and published Mosquito nodavirus and Yongsan tombus-like virus1 genome sequence (dark blue). Species in black were outgroup. **(D)** Small RNAs distribution across the genome of Mosquito nodavirus in both positive and negative stains. The x-axis (1 to 3000) represents the number of reads that cover each position of the genome. The y-axis is the count.

*Ae. albopictus* nodavirus was identified in one male pool. Most *Ae. albopictus* nodavirus mapped sRNA were 21 nt in length (**Figure 3B**), indicating that they were generated by the siRNA pathway. Four assembly contigs (**Figure 3A, D**) were generated, covering 43.2% of the genome with an average identity of 84.92%. The phylogenetic tree (**Figure 3C**) indicated that it was closely related to Mosquito nodavirus MNV-1 and Yongsan tombus-like virus 1.

### Hubei chryso-like virus 1

**Figure 4:**
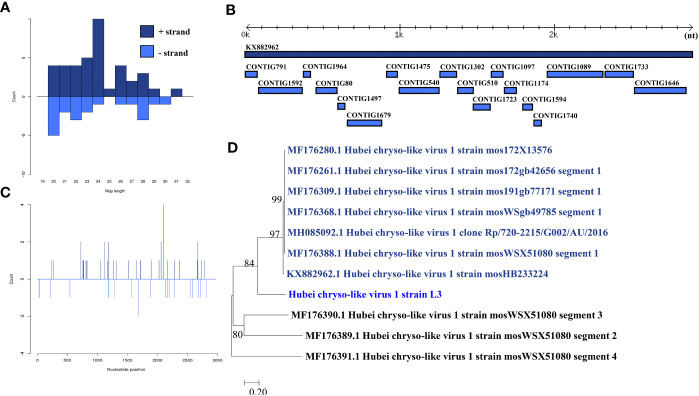
Analysis of small RNAs derived from Hubei chryso-like virus 1 and the phylogenetic analysis. **(A)** Alignment of assembly contigs to Hubei chryso-like virus 1 (KX882962) genome. Dark blue represents Hubei chryso-like virus 1 genome, and light blue represent assembled contigs. **(B)** Size distribution of sRNA derived from Hubei chryso-like virus 1. **(C)** Phylogenetic analysis indicates the relationship between the eight strains (light blue) and published Hubei chryso-like virus 1 genome sequence (dark blue). Species in black were outgroup. **(D)** Small RNAs distribution across the genome of Hubei chryso-like virus 1 in both positive and negative stains. The x-axis (1 to 3000) represents the number of reads that cover each position of the genome. The y-axis is the count.

Hubei chryso-like virus 1 was identified in one larva pool. Most Hubei chryso-like virus 1 mapped sRNA were 24 nt in length (**Figure 4B**), indicating that they were generated by the miRNA pathway. Eighteen assembly contigs (**Figure 4A, D**) were generated, covering 88.7% of the genome with an average identity of 98.33%. The phylogenetic tree (**Figure 4C**) indicated that it is closely related to Hubei chryso-like virus 1 Segment 1.

### *Aedes* flavivirus

**Figure 5:**
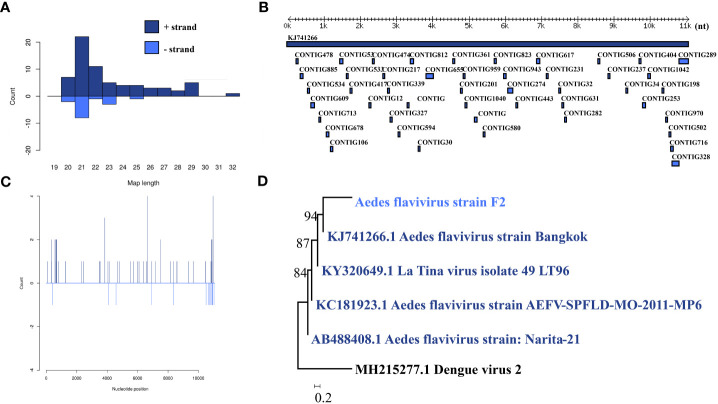
Analysis of small RNAs derived from AEFV and the phylogenetic analysis. **(A)** Alignment of assembly contigs to AEFV (KJ741266) genome. Dark blue represents AEFV genome, and light blue represent assembled contigs. **(B)** Size distribution of sRNA derived from AEFV. **(C)** Phylogenetic analysis indicates the relationship between the eight strains (light blue) and published AEFV genome sequence (dark blue). Species in black were outgroup. **(D)** Small RNAs distribution across the genome of AEFV in both positive and negative stains. The x-axis (1 to 10000) represents the number of reads that cover each position of the genome. The y-axis is the count.

*Aedes* flavivirus was identified in a female pool. Most *Aedes* flavivirus mapped sRNA were 21 nt in length (**Figure 5B**), indicating that they were generated by the siRNA pathway. Forty-seven assembly contigs (**Figure 5A, D**) were generated, covering 28.7% of the genome with an average identity of 97.87%. The phylogenetic tree (**Figure 5C**) indicated that it is closely related to Aedes flavivirus strain Bangkok.

### Tobacco rattle virus

**Figure 6:**
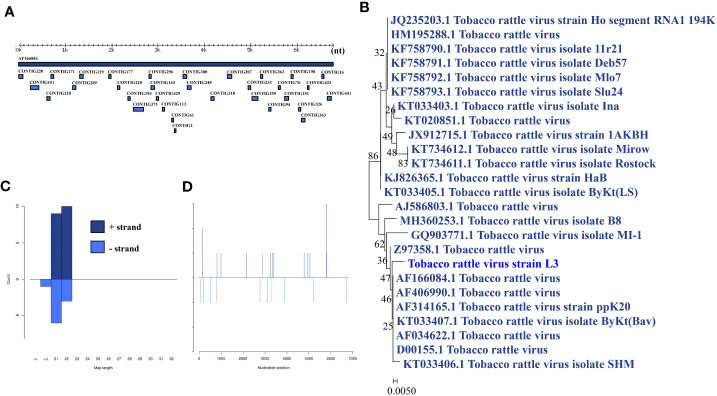
Analysis of small RNAs derived from tobacco rattle virus and the phylogenetic analysis. **(A)** Alignment of assembly contigs to tobacco rattle virus (AF166084) genome. Dark blue represents tobacco rattle virus genome, and light blue represent assembled contigs. **(B)** Size distribution of sRNA derived from tobacco rattle virus. **(C)** Phylogenetic analysis indicates the relationship between the eight strains (light blue) and published tobacco rattle virus genome sequence (dark blue). Species in black were outgroup. **(D)** Small RNAs distribution across the genome of tobacco rattle virus in both positive and negative stains. The x-axis (1 to7000) represents the number of reads that cover each position of the genome. The y-axis is the count.

Tobacco rattle virus was identified in a larva pool. Most tobacco rattle virus-mapped sRNA were 21-22 nt in length (**Figure 6B**), indicating that they were generated by the siRNA pathway. Thirty-two assembly contigs (**Figure 6A, D**) were generated, covering 32.8% of the genome with an average identity of 99.64%. The phylogenetic tree (**Figure 6C**) indicated that it is closely related to tobacco rattle virus.

### Contig assembly and identification of new viruses

In addition, several contigs with low identity to known viruses were discovered and are listed in **Table 5**. A number of contigs aligned with low identity to the amino acid sequence of Wuhan Mosquito virus 2 were identified in female pools (Female 1 and Female 2), male pools (Male 3), and larvae (Larvae 1 and Larvae 2). Four potential novel viruses were found in both female and male pools, which were closely related to Newfield virus (Female 1, Female 2, Female 3, Male 1, and Male 3), Hubei mosquito virus 2 (Female 1, Female 3 and Male 3), Diaphorina citri-associated C virus (Female 1, Female 2, Female 3, and Male 2), and Hubei permutotetra-like virus 3 (Female 2, Female 3, Male 1, Male 2, and Male 3). Nine potential novel viruses were found only in male pools, of which two were closely related to plant viruses: soybean leaf-associated ssRNA virus 1 and rice dwarf virus. Five potential novel viruses were found only in larvae pools, of which two were closely related to plant viruses.

**Table 5:** Assembly contigs associated with novel virus.

Segment Length	%Identity	Description	Discovery pool
1003	43.73	Newfield virus	Female 1, Female 2, Female 3, Male 1
497	56.87	Hubei mosquito virus 2	Female 1, Female 3, Male 3
398	22.67	Diaphorina citri associated C virus	Female 1, Female 2, Female 3, Male 2
389	41.45	Wuhan Mosquito Virus 2	Female 1, Female 2, Male 3, Larvae 1, Larvae 2
644	45.26	Wuhan Mosquito Virus 2	Female 1, Female 3
1030	40.15	Wuhan house centipede virus 9	Female 1
1238	21.8	Torrey Pines virus	Female 1
199	30.52	Hubei permutotetra-like virus 3	Female 2, Female 3, Male 1, Male 2, Male 3
603	31.25	Wuhan insect virus 21	Male 2
278	32.88	Whenzhou Shrimp Virus 2	Male 2
394	59.72	Hubei diptera virus 15	Male 2
199	39.24	Hubei permutotetra-like virus 3	Male 2, Male 3
553	61.78	Wuhan insect virus 35	Male 2
476	64.04	Hubei mosquito virus 4	Male 3
369	84.54	Mosquito nodavirus	Male 3
589	99.15	Wenzhou sobemo-like virus 4	Larvae 1
681	50	Wuhan Mosquito Virus 1	Larvae 1
102	30	Hepatitis E virus	Larvae 1
801	29.59	Rice dwarf virus	Larvae 2
1460	25	Homalodisca vitripennis reovirus	Larvae 2
531	34.9	Soybean leaf-associated ssRNA virus 1	Male 3
1019	19.29	Rice dwarf virus	Male 3

### Field survey of detected virus

**Figure 7:**
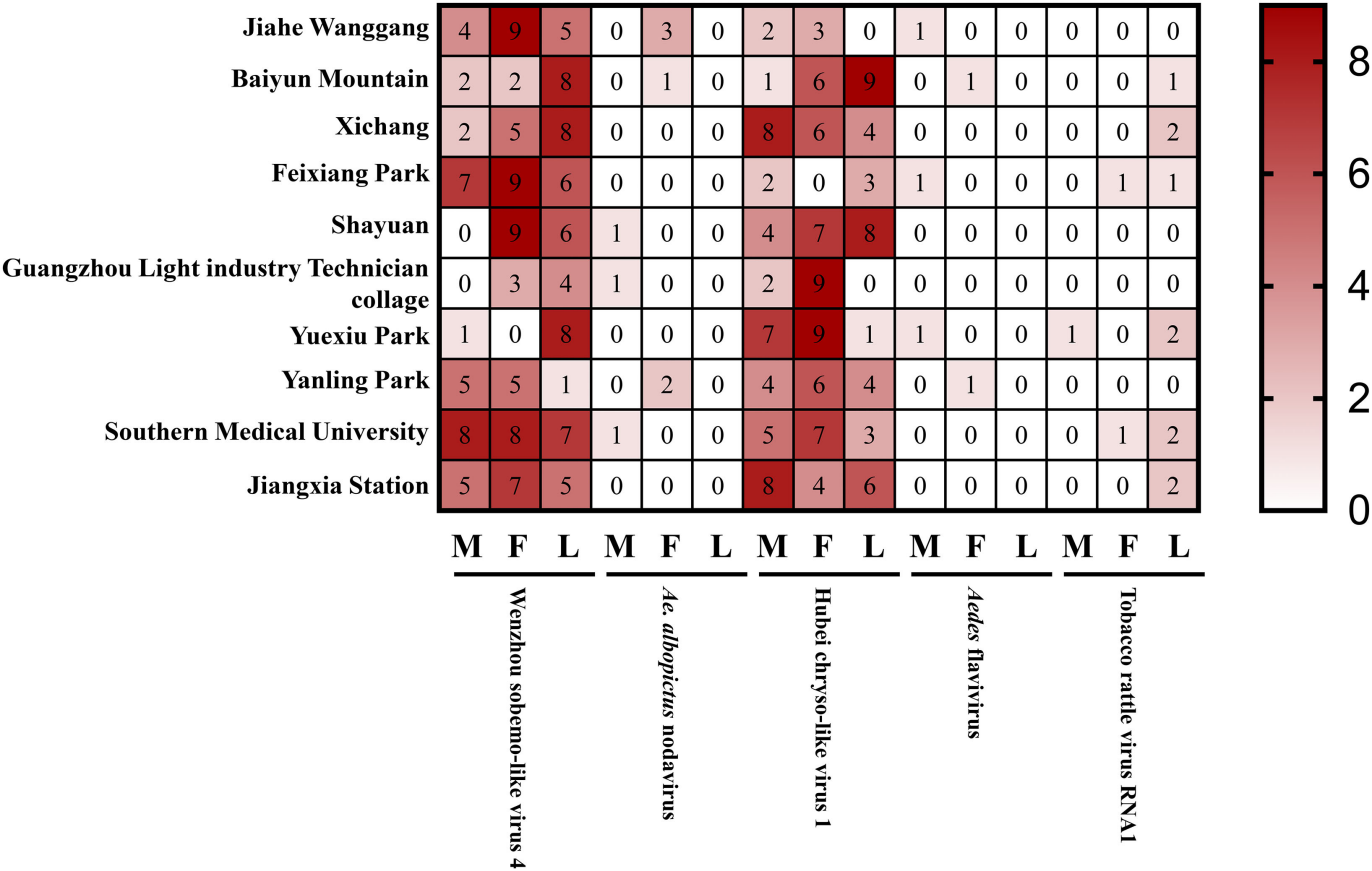
Detected viruses in wild *Ae. albopictus*.

To further study the detection of the above-mentioned viruses in *Ae. albopictus* collected from Guangzhou province, 10 unique specimens each of male and female adult and larvae were collected from various regions and detected by RT-PCR. The results showed that the highest detection rates were for Wenzhou sobemo-like virus 4 and Hubei chryso-like virus 1, which were found in both adult and larvae specimens (**Figure 7**). In contrast, detection rates for *Ae. albopictus* nodavirus, *Aedes* flavivirus, and tobacco rattle virus RNA1 were relatively low, with the latter two viruses only detected in adult mosquitoes. The results highlight the significance of further investigation into these viruses and their potential impact on public health.

## Discussion

*Ae. albopictus* is known to be a natural reservoir for many viruses transmitted by invertebrate vectors. This species is capable of transmitting several public health pathogens ((Kraemer et al., 2015), yet the full extent of its virome in the field remains unknown. In this study, we collected *Ae. albopictus* at different developmental stages and locations in Guangzhou, including parks, traffic stations, suburbs, schools, and office areas. We utilized small RNA sequencing to uncover the viral diversity and virus-associated sequences in *Ae. albopictus* from Guangzhou.

Our research shows that the majority of vsiRNAs for Wenzhou sobemo-like virus 4, mosquito nodavirus, *Aedes* flavivirus, and tobacco rattle virus were 21 nt in length, indicating that these viruses induced the siRNA pathway in *Ae. albopictus* after infection. Some reports suggest that virus infection can also activate the piRNA pathway in *Ae. albopictus*, leading to the production of piRNAs that are 25-29 nt in length (Miesen et al., 2016a; Miesen et al., 2016b; Joosten et al., 2019). In our study, a large number of Hubei chryso-like virus-specific small RNAs of 25-29 nt in length were detected, but the peak was at 24 nt, which is characteristic of miRNA production. Both miRNAs and piRNAs are classes of Dicer-independent small RNAs found in vertebrates and invertebrates, and they are believed to play a crucial role in maintaining genome stability in cells by targeting transposons (Varjak et al., 2017). However, the Hubei chryso-like virus-associated sequence was not found in the *Ae. albopictus* genome.

It is known that both female and male *Ae. albopictus* feed on plant nectar, and many plant-infecting viruses are transmitted by insect vectors. Our results showed that one known plant virus was detected in a larva sample, although it remains to be further studied whether it can be propagated. Plant virus transmission modes can be divided into four basic types: non-persistent, semi-persistent, persistent-circulative, and persistent-propagative. Some viruses can bind to specific insect vector cuticular locations without entering cells (noncirculative), while others can enter the insect gut and circulate or replicate within the insect vector body (Whitfield et al., 2015). According to this, only those viruses that enter the cell can trigger the RNAi pathway, so this method can only detect persistent-circulative and persistent-propagative viruses. However, some plant virus-associated contigs were detected in BLASTx, which requires further exploration.

*Aedes* flavivirus is a member of the *Flavivirus* genus in the Flaviviridae family, with a genome length of 11079 nt. It was first isolated in Japan in 2003 (Hoshino et al., 2009) and has since been recorded in Bangkok (Bolling et al., 2015) and Shanghai (Fang et al., 2018). It is a mosquito-borne flavivirus with unknown pathogenic capacity that has been isolated worldwide in natural mosquito populations. In this study, we identified an *Aedes* flavivirus that shares 97.87% identity with the known virus. We also identified Mosquito *Nodavirus*, which belongs to the Nodaviridae family. Mosquito *Nodavirus* was first identified in 2010 (Wu et al., 2010) using small RNA sequencing, but further research on this virus is still needed. We also detected two unclassified known viruses. The prevalence of Wenzhou sobemo-like virus 4 was reported in samples from *Culex quinquefasciatus* and *Culex tritaeniorhynchus* (Shi et al., 2015), and it was detected in female, male, and larvae samples in our study, suggesting possible vertical transmission. We also found several contigs related to known viruses with low identity, indicating the potential presence of novel viruses. However, these findings have yet to be verified, and the investigation of the city-wide white *Aedes* mosquito virome will be expanded upon in future studies. It is possible that the viruses identified in this study have a dietary or environmental origin, and the assembled sequences may be derived from food remnants. While the identified known virus contigs were insect-specific viruses not reported in any mosquito source organisms, we cannot entirely rule out the possibility that the contigs originated from the contamination of food remnants. Further research is needed to determine the origins of these novel viruses.

In field survey, we find the high detection rates of Wenzhou sobemo-like virus 4 and Hubei chryso-like virus 1 are particularly noteworthy, as their potential impact on public health is still uncertain. Therefore, the high detection rates of these viruses in mosquitoes suggest that further investigation is necessary to understand their potential impact on public health. The low detection rates for *Ae. albopictus* nodavirus, *Aedes* flavivirus, and tobacco rattle virus RNA1 suggest that these viruses are less prevalent in the mosquito populations of Guangzhou province. This could be due to several factors, including the season of mosquito collection, the location of collection, the sensitivity of the RT-PCR assay used to detect the virus, and the prevalence of the virus in the natural host population. It is possible that these viruses are more prevalent in other regions or mosquito populations, and further investigation is necessary to determine their distribution and prevalence.

One limitation of the study is the small sample size, which may limit the generalizability of the results. Additionally, the study only tested for a limited number of viruses, and there may be other viruses present in the mosquito populations that were not tested. Future research should expand the number of viruses tested and increase the sample size to provide a more comprehensive understanding of the distribution and prevalence of viruses in mosquito populations.

In conclusion, this study provides a valuable glimpse into the small RNA profiles of different sex and larvae of *Ae. albopictus* at different developmental stages and in various locations within one city. The identification and reconstruction of the mosquito virome revealed a diverse range of viruses, including several novel ones. However, given the limited sample size and the fact that only one mosquito species was collected, it is likely that this study represents only a small fraction of the overall mosquito virome. Nevertheless, the results shed light on the ecology and evolution of mosquito-borne viruses and highlight the importance of continued surveillance for potential threats to public health.

## Data availability statement

The datasets presented in this study can be found in online repositories. The names of the repository/repositories and accession number(s) can be found below: https://www.ncbi.nlm.nih.gov/genbank/, PRJNA527345.

## Author contributions

X-GC conceived and contributed to the study concept and design. YX, JX, TL and PL collected the samples. YX and JX completed all analyses and finished the manuscript. X-GC supervised the study. All authors contributed to the article and approved the submitted version.
